# Supplementary material for: Silica Particles Trigger the Exopolysaccharide Production of Harsh Environment Isolates of Growth-Promoting Rhizobacteria and Increase Their Ability to Enhance Wheat Biomass in Drought-Stressed Soils
Source: Int J Mol Sci. 2021 Jun 8;22(12):6201. doi: 10.3390/ijms22126201 (PMC8229586; doi:10.3390/ijms22126201)
Supplement: Supplementary file 1 [file ijms-22-06201-s001.zip › supplementary/ijms-1184279-proofed-supplementary .pdf]

## Supplementary Materials

# Silica Particles Trigger the Exopolysaccharide Production of Harsh Environment Isolates of Growth-Promoting Rhizobacteria and Increase Their Ability to Enhance Wheat Biomass in Drought-Stressed Soils

**Anastasiia Fetsiukh [1], Julian Conrad [2], Jonas Bergquist [3], and Salme Timmusk [1,4,*]**

[1] Department of Forest Mycology and Plant Pathology, Swedish University of Agricultural Sciences (SLU) Uppsala, PO 7026, SE 75007, Sweden; stimmusk@hotmail.com
[2] Swedish National Cryo-EM Facility, Science for Life Laboratory, Sweden PO 1031, SE 171 21 Sweden; julian.conrad@scilifelab.se
[3] Department of Chemistry, Uppsala University, PO 576, SE 75123 Sweden; jonas.bergquist@kemi.se
[4] Bashan Institute of Science, 1730 Post Oak Ct, Auburn, AL 36830, USA
* Correspondence: salme.timmusk@slu.se

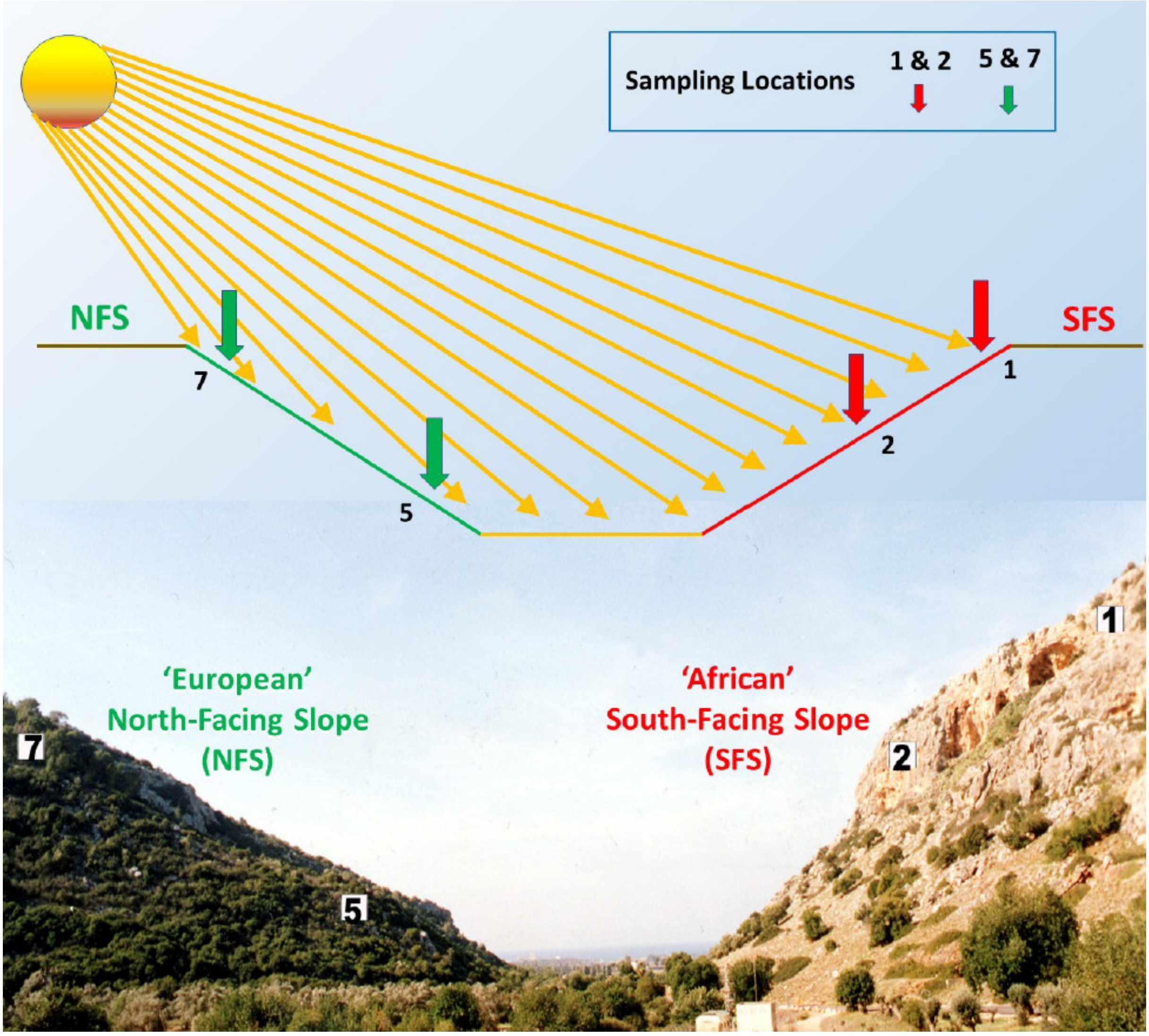


**Figure S1.** The Evolution Canyon (EC) model: Schematic diagram of the Evolution Canyon at Lower Nahal Oren, Mount Carmel (source[10]: Nevo, 2012 Evolution Canyon," a potential

microscale monitor of global warming across life, PNAS 109; 8) (Photo by S. Timmusk).

**Table S1. Strains used in the study**

| Name | Wild barley rhizosphere, the Evolution Canyon, Haifa, Israel | Publications |
|---|---|---|
| *Paenibacillus polymyxa* A26 | Wild barley rhizosphere, the Evolution Canyon, Haifa, Israel | [1] |
| *Paenibacillus polymyxa* A26Sfp | Wild barley rhizosphere, the Evolution Canyon, Haifa, Israel | [2–4] |

## Video S1

A. Material Typical performance of A26 and A26Sfp; B. A26SN and A26SfpSN. See Material and Methods.

## Reference


1. Timmusk, S.; Paalme, V.; Pavlicek, T.; Bergquist, J.; Vangala, A.; Danilas, T.;Nevo, E. Bacterial distribution in the rhizosphere of wild barley under contrasting microclimates. *PLoS One*. **2011**, *6*, 1–8, doi: 10.1371/journal.pone.0017968.
2. Timmusk, S.; Kim, S.-B.; Nevo, E.; Abd El Daim, I.; Ek, B.; Bergquist, J.;Behers, L. Sfp-type PPTase inactivation promotes bacterial biofilm formation and ability to enhance wheat drought tolerance. *Front. microbiol.* **2015**, *6*, 387, doi: 10.3389/fmicb.2015.00387.
3. Abd El-Daim, I.; Haggblom, P.; Karlsson, M.; Stenstrom, E.; Timmusk, S. *Paenibacillus polymyxa* A26 Sfp-type PPTase inactivation limits bacterial antagonism against *Fusarium graminearum* but not of *F. culmorum* in kernel assay. *Front. Plant Sci*. **2015**, 6, 368. doi: 10.3389/fpls.2015.00368.
4. Kim, S.-B.; Timmusk, S. A simplified method for *Paenibacillus polymyxa* gene knockout and insertional screening. *PLoS One* **2013**, *8*, e68092. doi: 10.1371/journal.pone.0068092.
